# Flavopiridol (Alvocidib), a Cyclin-dependent Kinases (CDKs) Inhibitor, Found Synergy Effects with Niclosamide in Cutaneous T-cell Lymphoma

**DOI:** 10.33696/haematology.2.028

**Published:** 2021-05-04

**Authors:** Xu Hannah Zhang, Jack Hsiang, Steven T. Rosen

**Affiliations:** Department of Hematology and Hematopoietic Cell Transplantation, City of Hope, Beckman Research Institute, National Medical Center, Duarte, CA 91010, USA

**Keywords:** Flavopiridol, p38γ, ATP binding pocket, NF-κB pathway, PamGene platform, Kinomic profiling, Development of synergistic drug

## Abstract

Flavopiridol (FVP; Alvocidib), a CDKs inhibitor, is currently undergoing clinical trials for treatment of leukemia and other blood cancers. Our studies demonstrated that FVP also inhibited p38 kinases activities with IC_50_ (μM) for p38α: 1.34; p38 β: 1.82; p38γ: 0.65, and p38δ: 0.45. FVP showed potent cytotoxicity in cutaneous T-cell lymphoma (CTCL) Hut78 cells, with IC_50_ <100 nM. NMR analysis revealed that FVP bound to p38γ in the ATP binding pocket, causing allosteric perturbation from sites surrounding the ATP binding pocket. Kinomic profiling with the PamGene platform in both cell-based and cell-free analysis further revealed dosage of FVP significantly affects downstream pathways in treated CTCL cells, which suggested a need for development of synergistic drugs with FVP to prevent its clinically adverse effects. It led us discover niclosamide as a synergistic drug of FVP for our future *in vivo* study.

## Introduction

Flavopiridol (FVP; alvocidib), an FDA-approved orphan drug, has been studied in clinical trials under both single treatment and combination scenarios; several single-agent Phase I and Phase II clinical trials against leukemia, lymphomas, and solid tumors are active [1–3]. To date, there have been more than 50 clinical trials involving FVP in the United States [4]. Unfortunately, almost half of patients on FVP clinical trials showed serious adverse effects, implicating appropriate dosages need to be found and an alternative way to circumvent the toxicity of FVP with synergistic agents. Here, we summarize a selection of completed studies that had posted results in Table 1.

Currently four FVP trials are actively recruiting, two in patients with AML (NCT03969420, NCT03441555), one in patients with myelodysplastic syndrome (NCT03593915), and one in patients with solid tumors (NCT03604783). However, there has yet to be any publication or clinical trial focusing on the effects of FVP on Cutaneous T cell lymphoma (CTCL) which is a disfiguring and incurable cancer with limited effectual therapeutic options. Here, we report that the CDK inhibitor FVP also inhibits p38 kinase activity and induces apoptosis in cultured CTCL cells in the nanomolar range. The purpose of this study was to determine the mechanisms through which FVP-mediated inhibition of signaling molecules induces cell death in CTCL, which will have far reaching significance toward the cure of this disease.

## Results

### FVP targets p38γ kinase activity in a cell-free based assay

**Figure 1:**
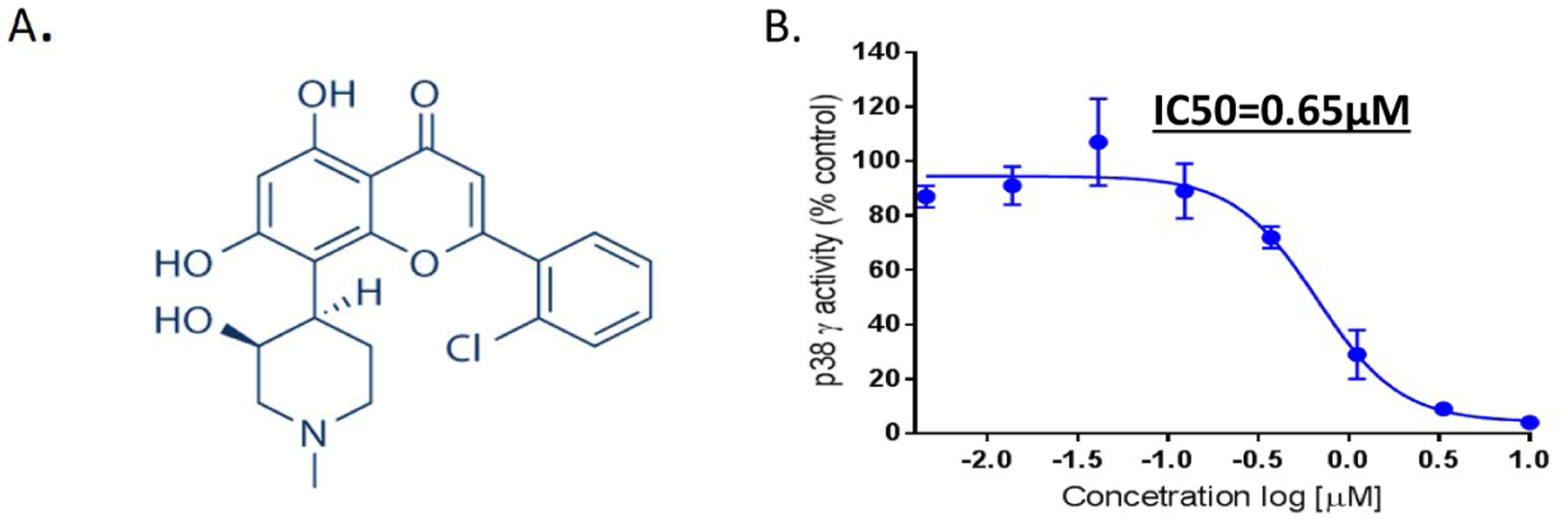
FVP inhibits p38γ kinase activity. (**A**) Structure of FVP; (**B**) IC_50_ of FVP against enzyme activity of p38γ isoform.

We have identified one important signaling molecule, the mitogen-activated protein kinase p38γ, which is overexpressed in CTCL cells, but not in normal healthy T cells [5]; this makes it a good target for developing a drug against CTCL. Therefore, we attempted to screen p38γ inhibitors from a natural product library by performing an *in vitro* kinase assay using ADP-Glo as readout for activated p38 isoforms. We tested a few flavonoid-backbone compounds for their anti-p38 activities (Appendix A. Figure 1A). We noted only FVP and myricetin have anti-p38 kinase activities. Myricetin targets three isoforms except p38δ, the IC_50_s are p38α: 1.34 μM; p38β: 1.82 μM and p38γ: 1.6 μM.

In Figure 1A, we demonstrate that FVP (with flavo-backbone structure) inhibits p38γ kinase activity with an IC_50_ of 0.65 μM (Figure 1B). We further analyzed FVP inhibition of all other isoforms of p38 (α, β, and δ) and found the IC_50_s to be p38α: 1.34 μM; p38β: 1.28 μM; and p38δ: 0.45 μM (Appendix A. Figure 1B).

**Figure 2:**
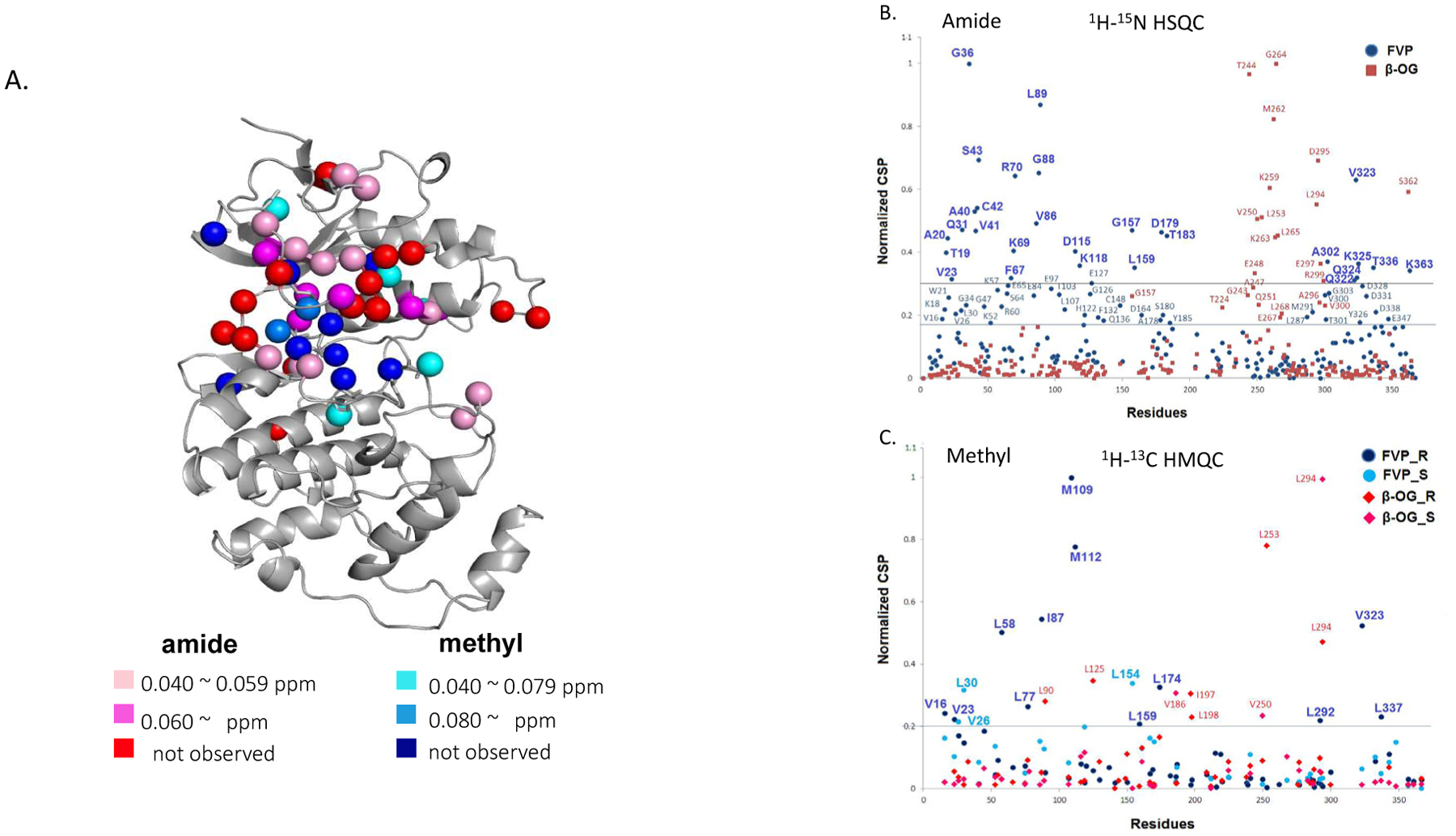
FVP binds to ATP binding pocket of p38γ which has allosteric binding outside the pocket (**A**) 3D structure of p38γ chemically perturbed by FVP based on 2D NMR CSP experimental results; (**B**) normalized NMR CSP ^1^H-^15^N data for p38γ with FVP (round blue dots) and β-OG; (**C**) normalized NMR CSP ^1^H-^13^C data for p38γ with FVP (round blue dots) and β-OG (red square or diamond), respectively.

To confirm the specificity of FVP for p38γ, we performed NMR titration and identified the FVP binding site on p38γ. Extensive NMR chemical shift changes and line broadening were observed in both 1H-15N HSQC and 1H-13C HMQC spectra upon the addition of FVP (Appendix A. Figure 2). Residues with line broadening effects are indicated in red (amide) and blue (methyl) on the p38γ structure (Figure 2A); other residues with large NMR chemical shift perturbations (CSPs; calculated as described in Methods) are represented in different colors according to the categories shown. The most significant chemical shift changes occurring around the ATP-binding pocket infer that FVP associates with p38γ in the pocket.

We further analyzed normalized CSP of FVP in comparison to β-OG, a small lipid molecule that binds to the lipid binding site of p38γ (unpublished data). We confirmed allosteric binding because CSPs propagated to residues distal from the ATP-binding site, mostly in the N-lobe, caused by the binding of FVP such as G36, A40, K69, L89 and K363 (Figure 2A). The residues that lie outside the ATP pocket according to normalized CSPs are: M109, M112, D116, K118, L154, G157, L159, L174, L292, A302, K325, V323, L334, T336 and L337 (line broadening or CSP > 0.02–0.03 ppm in amide/methyl correlation spectra) (Figure 2B and 2C). This suggests that the allosteric effects of FVP at high dosages on the kinase activity of p38γ, which may apply to other p38 isoforms, probably causing adverse effects when all four isoforms of p38 are inhibited *in vivo*.

### FVP has cytotoxic effects on Hut78 cells compared to other flavonoid compounds.

**Figure 3:**
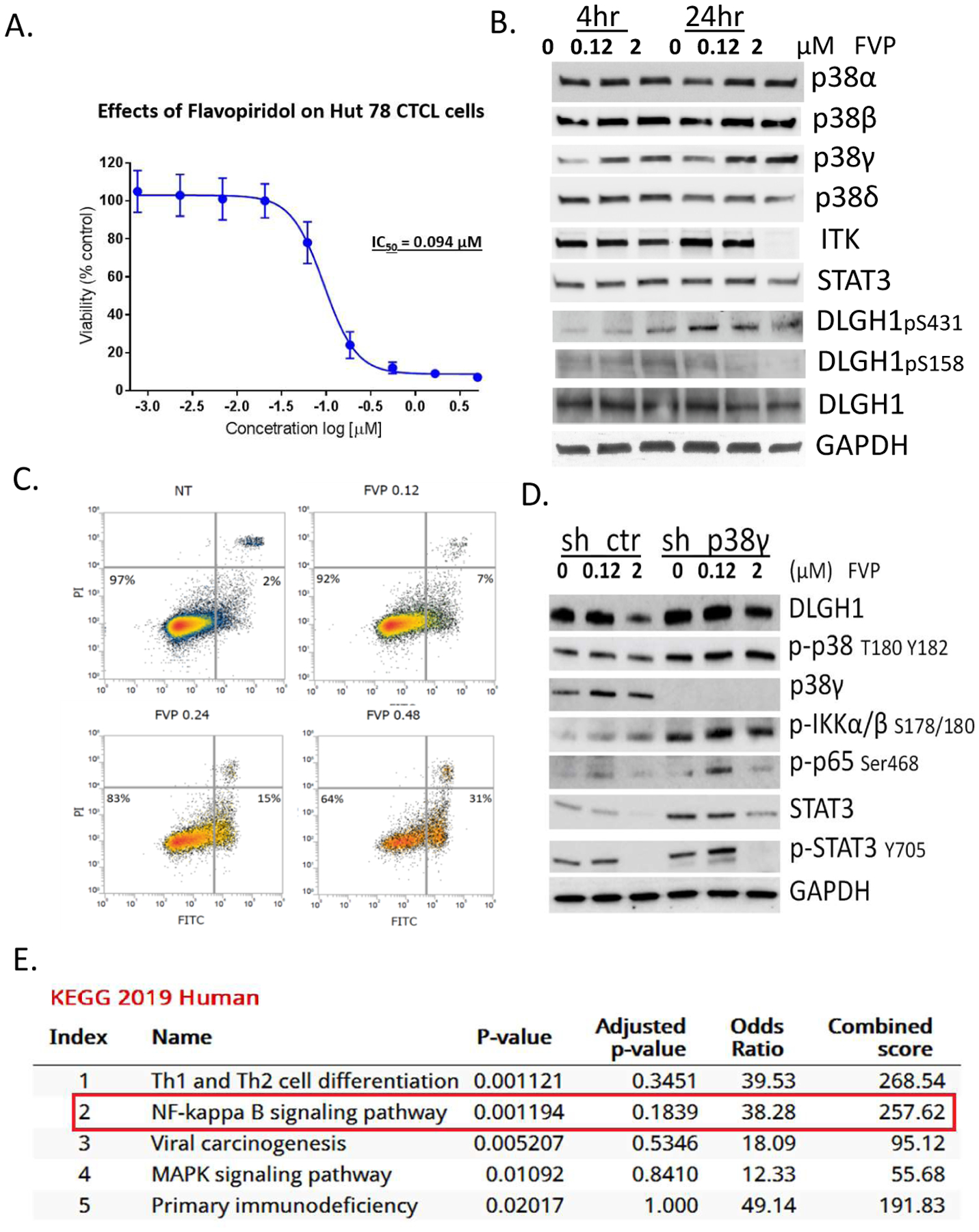
Cytotoxicity IC_50_ measurement of FVP in Hut 78 cells. (**A**) IC_50_ of FVP in Hut78 CTCL cells; (**B**) Western blot results of Hut78 cells treated with 0, 0.12, or 2 μM of FVP for 4 or 24 hours; (**C**) FVP induces apoptosis in Hut78 cells. Annexin V apoptosis assay of Hut78 cells treated with 0, 0.12, 0.24, or 0.48 μM FVP for 48 hours; (**D**) Loss of p38γ increases differential cytotoxicity effects by dosage difference. Western blot of shCtr and shp38γ cells treated with 0, 0.12, or 2 μM FVP for 24 hours; and (**E**) Pathway analysis with KEGG 2019 human pathway indicating NF-κB and MAPK are top pathways targeted by FVP in p38γ-silenced Hut78 cells.

Given that FVP inhibits p38γ kinase activity, and that p38γ is elevated in CTCL cells and is important for cell viability [5], we tested the cytotoxic effects of FVP on Hut78 CTCL cells. FVP was cytotoxic to Hut78 cells, with IC_50_ = 0.094 μM (Figure 3A), which is far more potent compared to any natural flavonoid compounds (Appendix A. Figure 3). Cytotoxicity IC_50_ of myricetin for Hut78 cells is 19.2 μM.

To understand molecular mechanism of FVP in Hut78 cells, we performed Western blot analysis of Hut78 cells treated with 0.12 μM or 2 μM FVP and showed that within 4 hr, p38α and p38δ protein expression did not change significantly, whereas p38γ expression increased with dose (Figure 3B). Within 24 hr, ITK expression disappeared upon 2 μM FVP treatment. Further reduction of pSTAT3 Y705 indicated cell cytotoxicity is due to targeting STAT3 (Figure 3D). The reduction of DLGH1 pS158 suggests blockage of the nuclear factor of activated T-cells (NFAT) pathway upon FVP treatment by 24 hr and it is partially due to p38γ inhibition in Hut78 cells, because DLGH1 pS158 is specifically phosphorylated by p38γ and activated DLGH1 directs TCR signaling in the NFAT pathway, as we and others previously reported [5,6].

To investigate whether FVP induced apoptosis in Hut78 cells, we performed Annexin V staining after treatment. Indeed, FVP caused apoptosis in Hut78 cells (Figure 3C, the apoptosis population increased with dose from 7% to 31%). Thirty-one percent of Hut78 cells treated with 240 nM FVP for 48 hr are Annexin V-positive, which indicates cells that have gone through apoptosis.

To further confirm the mechanisms through which FVP-mediated cell death is due to or partially due to inhibition of p38γ, we knocked down p38γ using a lenti-viral shRNA strategy, followed by Western blot analysis (Figure 3D). We found loss of p38γ increases the contrast of cytotoxicity effects by two different dosages, i.e., 120 nM and 2 μM in sh_p38γ-treated Hut78 cells, compared to control cells (shCtr). Upon 0.12 μM FVP for 24 hr treatment, several signaling proteins were upregulated such as NF-κB related signal proteins p65 Ser468, pIKKα/β Ser178/180, and p-STAT3 Y705 and STAT3, but downregulated by 2 μM FVP treatment, suggesting p38γ participates in the NF-κB pathway in FVP-induced apoptosis at high-doses (Figure 3D).

We further performed PamGene kinomic profiling analysis following FVP (120 nM) in comparison to p38γ-silenced Hut78 cells (KEGG human 2019). We showed that p38γ participated in Th1 and Th2 cell differentiation, the NF-κB pathway and MAPK pathway (Figure 3E), which confirmed our Western blot results that p38γ plays an indispensable role in the FVP-triggered NF-κB pathway.

### Protein kinomic profiling of FVP-treated Hut78 cells and p38γ gene silencing Hut78 cells with PamGene platform

**Figure 4:**
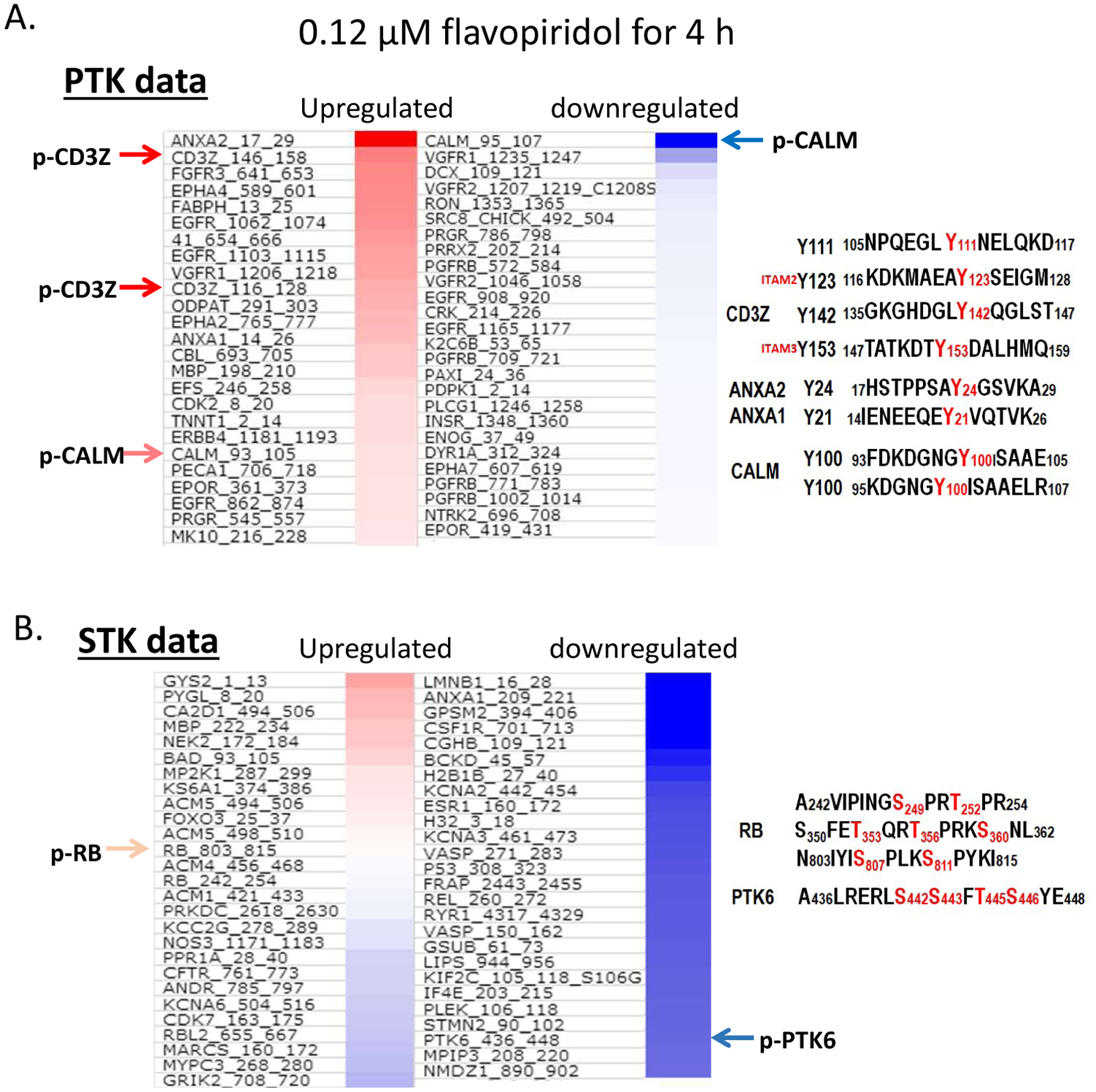
Kinomic profiling with PamGene in FVP-treated Hut78 cells. (**A**) Protein tyrosine kinase (PTK) assay; (**B**) Serine-threonine kinase (STK) assay.

To understand functionally relevant properties of kinases alteration in CTCL cells by FVP, we performed PamGene kinomic profiling in Hut78 cells. The PamGene platform revealed a few selective downstream targets with differential expression upon FVP treatment. Using a protein tyrosine kinase (PTK) assay (Figure 4A), we measured protein tyrosine kinase activities in Hut78 cells treated with 120 nM FVP for 4 hr. We identified tyrosine phosphorylation of Calmodulin, Annexin A2/A1 and CD3ζ are significantly changed. Serine and Threonine kinase (STK) assays were also applied, and our results showed that many downregulated targets by FVP at 4 hr aligned with the NF-κB pathway (Figure 4B).

#### a) PTK panel assay identified phosphorylation of Calmodulin at Y100 and phosphorylation of ITAM sites of CD3ζ

The PTK heatmap (Figure 4A) reveals that phosphorylation sites of each protein and their responses to FVP. Phosphorylation of both ANXA2 (aa17_29) and ANXA1 (aa14_26) showed increased, whereas phosphorylation of CALM (aa95_107) significantly decreased by FVP, compared to untreated control cells (Figure 4A, indicated with arrows). Tyrosine phosphorylation levels of both Tyr123 of CD3ζ (aa116_128) and Tyr158 of CD3ζ (aa146_158) are increased, which was further confirmed by our western blot analysis of p-Tyr antibody staining in Hut78 cells that most of proteins of which tyrosine phosphorylation are increased by FVP at 4 hr. With two dosages of FVP at 2 time-points, phosphorylation of tyrosine kinases is increased upon treatment at 4 hr in general, but it has significantly decreased at 24 hr of FVP treatments (Appendix A. Figure 4).

Here we focus on phosphorylation of Calmodulin at Y100 (p-Y100 CaM) for further discussion, because it is the only phosphorylation that showed significant decreases by FVP at 4 hr. The phosphorylation of human calmodulin at Y100 plays many vital pathological roles in cancer. It enhanced ligand-dependent EGFR activation in an in vitro study [7]. It binds to the SH2 domain of the regulatory p85 subunit of PI3K, activating the catalytic p110 subunit and the K-Ras/PI3K/Akt pathway. Molecular modeling suggests that direct interaction occurs between the p-Y100 CaM and PI3Kα with high affinity, which fully activates PI3Kα by oncogenic K-Ras to promote cancer [8–10]. One possible mechanism of FVP function in CTCL is to arrest cell cycle progression by dramatically reducing tyrosine phosphorylation on CaM at Y100 at its earlier time point of treatment to prevent its direct interaction with PI3Kα and thereby to halt cell growth. As to the curation of Y100 on calmodulin (^93^FDKDGNG**Y**_**100**_ISAAE^105^), until 2018, all but one citation [11], described it as Phospho-Y99-CaM (DGNG**Y**_**99**_ISAA) of human calmodulin [7–10,12–14]. Here we use p-Y100-CaM as it is currently curated in Uniport and phosphor Site, since the initiator methionine was removed [15].

Another important target protein identified by our PamGene analysis of FVP-treated Hut78 cells is CD3ζ, whose phosphorylation at Y123 (ITAM 2) and Y153 (ITAM 3) are increased (FVP 120 nM at 4 hr). CD3ζ, alternatively named as the T-cell receptor (TCR) zeta chain, is an essential TCR signaling protein. Shortly after TCR activation, the lymphocyte specific PTK Lck gains proximity to the TCR/CD3 complex and phosphorylates intracellular tyrosine activation motifs (ITAMs) within the CD3 complex. Phosphorylation of the CD3 ITAMs promotes the docking of ZAP70 to the CD3 complex, which is also phosphorylated by Lck for its activation [9,16,17].

Further analysis with p38γ gene silencing Hut78 cells (sh– p38γ) that are treated with FVP showed that loss of p38γ and/or inhibition of p38γ activity failed to phosphorylate ITAM 2 (Y123) or ITAM 3 sites (Y153) of CD3ζ. This suggests that loss of p38γ in Hut78 cells results in loss of high-affinity interaction between CD3ζ and ZAP70 when the phosphorylation at ITAM sites are annulled, as it is known that the phosphorylation on ITAM 2 or 3 sites of CD3ζ are crucial for ζ-associated protein of 70 kilodaltons (ZAP70) recruitment through high-affinity binding sites on CD3ζ [18]. CTCL Hut78 cells became malignant likely due to p38γ direct interaction with TCR complex proteins, which may address the reason why the normal healthy T cells keep p38γ silenced.

#### b) STK panel phosphorylation of Hut78 cells

**Figure 5:**
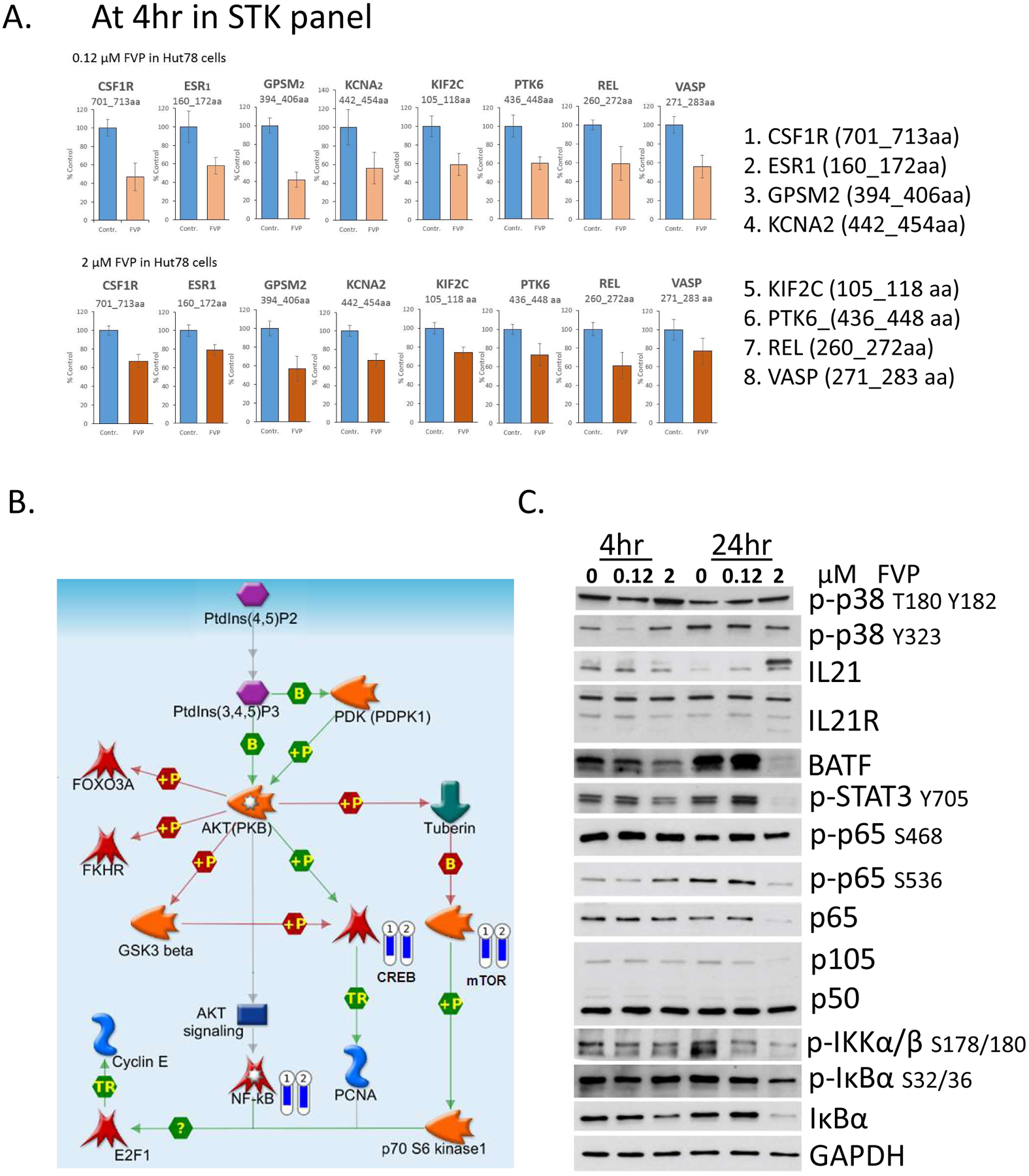
STK kinomic assay showed NF-κB is major targeted pathway by FVP in CTCL cells. (**A**) a list of target gene hit by FVP has NF-κB binding motif that are down-regulated in their phosphorylation; (**B**) Metacore pathway analysis of FVP treated Hut cells; (**C**) Western blot assessment of phospho-proteins of Hut78 cells treated with 0, 0.12, or 2 μM of FVP for 4 or 24 hours.

Using the serine-threonine kinase (STK) PameGene panel assay (Figure 4B), we screened downregulated Ser/Thr phosphorylation targets, and proteins with significant reduction of Ser/Thr phosphorylation were as follows: LMNB1, ANXA1, GPSM2, CSF1R, CGHB, BCKD, H2B1B, KCNA2, ESR1, H32, KCNA3, VASP, P53, FRAP, REL, RYR1, VASP, GSUB, LIPS, KIF2C, IF4E, PLEK, STMN2, PTK6, MPIP3, and NMDZ1. We scored the DNA sequences of above genes against the position weight matrix (PWM) with TRANSFAC and JASPAR database, and found binding motifs for NF-κB existed at the promoters of in the following genes: VASP, GPSM2, CSF1R, KCNA2, REL, PTK6, KIF2C, and ESR1. We further confirmed that phosphorylation level of each protein product of these genes was reduced by 120 nM FVP treatments at 4 hr in Hut78 cells (Figure 5A), which strongly support that FVP inhibits NF-κB pathway via multiple downstream target genes in Hut78 cells. The phosphorylation of REL/p65 at Ser267 (^260^KMQLRRP**S**_**267**_DQEVS^272^) also reduced by FVP echoes others’ finding that a direct link between MAPK and NF-κB pathways by MAPK downstream kinase Mitogen-and Stress-activated Kinase 1 (MSK1) -mediated phosphorylation of Ser276 REL/p65 of NF-κB [19].

In addition, the reduction in phosphorylation of p53 Ser315 (^308^LPNNTSS**S**_**315**_PQPKKKPLDGE^326^) in the STK panel by FVP treatment is also observed (Figure 4B). It suggests that FVP inhibits CDK1/2 [20] or/and AURKA [21] in Hut78 cells. In addition, Metacore pathway analysis showed the involvement of CREB and mTOR along the path targeting NF-κB by FVP (Figure 5B).

Of note another important regulator targeted by FVP is protein tyrosine kinase 6 (PTK6), the phosphorylation of which was significantly reduced at S442 ( ^436^ALRERLS_442_S_443_FT445S_446_Y^448^). PTK6, also known as breast tumor kinase (BRK), is a non-receptor kinase that is negatively regulated by phosphatases including protein tyrosine phosphatase 1B (PTP1B) and phosphatase and tensin homologue (PTEN) [22]. Many downstream targets of PTK6 have been identified, including RNA-binding proteins in the nucleus such as SAM68, and signaling molecules in the cytosol [23]. Notably, PTK6 substrates in the cytoplasm include transcription factors STAT3 and STAT5a/b, which are activated upon phosphorylation and translocate into the nucleus to promote gene expression [24,25]. We have observed reduction of PTK6 phosphorylation by PamGene experiment following 4 hr FVP treatment of Hut78 cells (Figure 5A), p-STAT3 Y705 was first upregulated at 4 hr and was reduced at 24 hr by FVP (Figure 5C), which suggests that PTK6 is an upstream regulator of STAT3 in CTCL.

#### c) Differential biological effects of lower dosage vs. higher dosage of FVP

Side effects of FVP in clinical usage prompt us to dissect the optimal dosage of FVP for CTCL. We compared two datasets of PamGene profiling at two FVP doses, 120 nM and 2 μM, and observed that 15 target proteins exhibited contrasting differential phosphorylation status: PTPN12, CREB1, RYR1, GRIK2, NCF1, KCNA6, PFKFB3, CFTR, VTNC, KAP3, EPB42, KIF2C, KPB1, SCN7A, and CSF1R. To identify the subcellular location of these 15 protein targets, we performed database analysis Jensen Compartments using the EnrichR program [26]. The top hits we identified are ion channel complex and transporter complexes (p<0.000001), which matches five targets on the 15-protein list, RYR1, GRIK2, KCNA6, CFTR, and SCN7A (Appendix A. Figure 5). We used Western blot analysis to assess important signaling proteins response to FVP treatment in Hut78 cells with 2 μM FVP for 24 hr, we observed unchanged phosphorylation on NF-κB p65 at Ser468, but phosphorylation on p65 at Ser536 is downregulated (Figure 5C). Ser536 is within the transactivation domain (TAD) of p65, and is a phosphorylation site that is targeted by several kinases, including IKKβ [27,28]; we also observed downregulation of both NF-κB subunits p65 and p105.

Combined with our apoptosis analysis at 48 hr (Figure 3C), we concluded that when FVP is above 240 nM, it increases apoptosis in Hut78 cells compared to that of lower doses (below 120 nM), which demarcates as high dosage cytotoxicity effects of FVP. The high-dose FVP caused damage to ion channels in T cells, most likely to the calcium channels (Appendix A. Figure 5).

### Selection of drugs that have synergistic effects with FVP

To develop novel drugs that work synergistically with FVP, we selected inhibitors of which signaling pathways are complementary to FVP p38γ inhibition, with an expectation that the combination treatments only kill cancer cells but spare healthy cells.

#### a) FVP in combination with the PTK6 inhibitor cpd 4f

**Figure 6:**
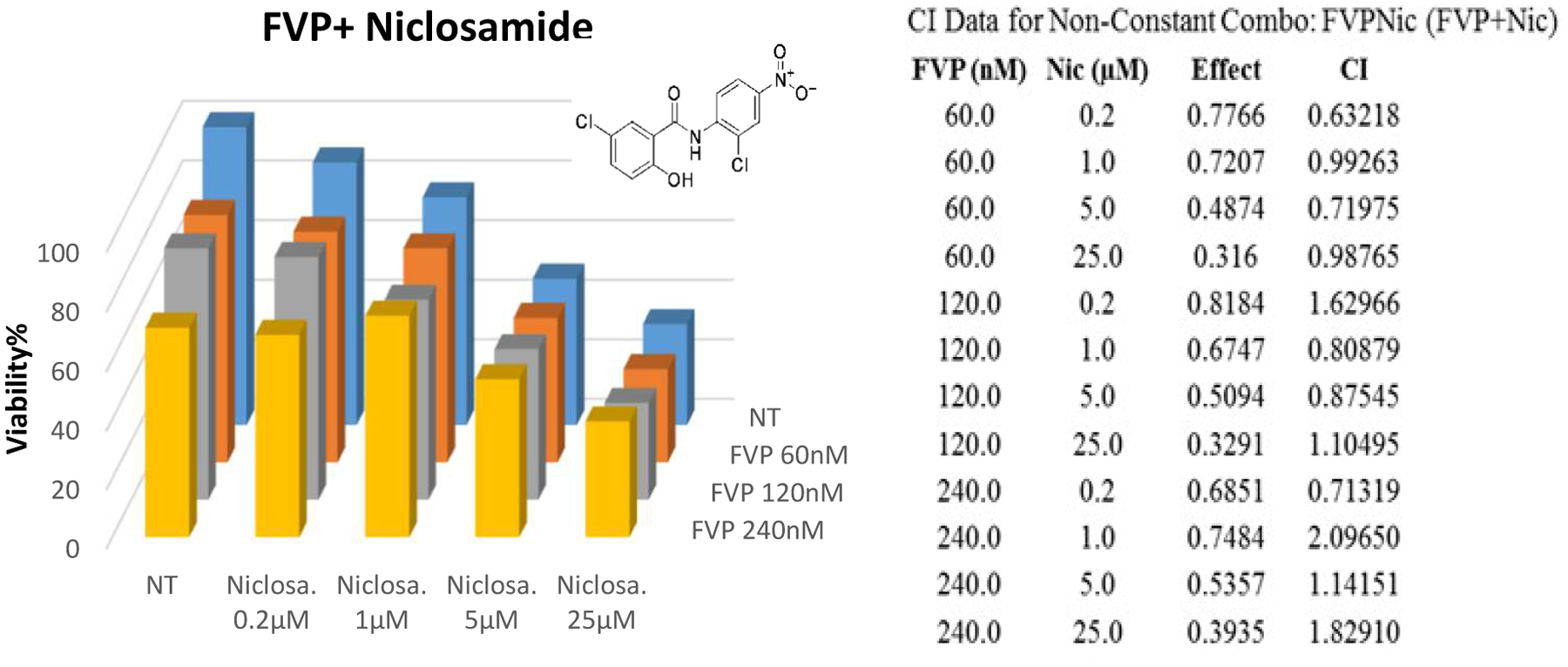
Combination treatment of FVP and niclosamide in Hut78 cells. (**A**) MTS results of Hut78 cells after combination treatment with FVP (0, 60nM, 120nM, or 240nM) and niclosamide (0, 0.2 μM, 1 μM, 5 μM, or 25 μM) for 72 hours; (**B**) Calculation of CI using CompuSyn.

Our observation that phosphorylation of the non-receptor tyrosine kinase PTK6 was significantly inhibited by FVP in our STK panel of kinomic profiling (PamGene) experiments (Figure 4B) supports our rationale for selecting PTK6 as a target. In addition, the STAT3–PTK6–NF-κB axis targeted by PTK6 inhibitors is very likely complementary to FVP efficacy through p38 inhibition. We tested this by selecting the PTK6-specific inhibitor cpd 4f for synergistic study with FVP. We treated Hut78 cells for 72 hr with both FVP and cpd 4f, using two doses of FVP (90 nM, 180 nM) and four doses of cpd 4f (15 nM, 30 nM, 15 μM, 30 μM) and measured cell viability to assess for drug synergy (Appendix A. Figure 6). Our results show no significant synergy in Hut78 cells using the combination treatment, but our future studies will involve testing additional PTK6-specific inhibitors to identify one that works synergistically with FVP. We also attempt to choose inhibitors that inhibit other pathways other than STAT3-NF-κB pathway.

#### b) FVP in combination with the dual STAT3 and mTOR inhibitor niclosamide

Niclosamide, a dual inhibitor of STAT3 and mTOR, was selected not only because of its efficacy against cancer and its synergistic effects with many other drugs [29]; but because the mTOR pathway is parallel to STAT3–NF-κB axis. Niclosamide is known to act synergistically in combination with many frontline chemotherapeutic agents in acute myelogenous leukemia (AML), including Ara-C (Cytarabine), VP-16 (etoposide), and DNR (daunorubicin) [30]. We tested its synergistic effects with FVP, which inhibit NF-κB–regulated genes [31]; whereas Niclosamide targets STAT3 and mTOR which served as our rationale of the complement treatment.

We treated Hut78 cells with three doses of FVP (60 nM, 120 nM, 240 nM) in combination with four doses of niclosamide (200 nM, 1 μM, 5 μM, 25 μM) for 72 hours to examine drug synergy. We found a synergy effect (CI=0.6) of niclosamide (200 nM) with FVP (60 nM). In addition, the combination of 200 nM niclosamide and 240 nM FVP also seemed to exhibit synergistic effects (CI=0.7) (Figure 6).

## Discussion

Niclosamide is an FDA-approved drug [31] that has been successfully used for over 5 decades to treat tapeworm infections with few adverse effects. Now it has found a new application in cancer therapy [29]. Its molecular mechanism has been thoroughly studied and occurs through targeting Wnt/β-catenin, mTOR, JAK/STAT3, NF-κB, and Notch pathways [32]. Based on our study, a higher dose FVP (240nM) impairs ion channel complexes of cancer patients likely contributed to the adverse effects that FVP recipients experienced in clinical trials. Our finding of synergistic effects of niclosamide with FVP has great potential for therapeutic application in CTCL and other hematopoietic cancers; the synergy allowed the dose of FVP to be reduced to 60 nM, the amount less likely to have adverse effects. The effective niclosamide dose was also reduced when used synergistically. We are currently performing *in vivo* studies of FVP and niclosamide synergy using Hut78 cell–xenograft and CTCL patient-derived xenograft mouse models. Here, we show that FVP inhibited CDK9 and p38γ, and showed potential clinical efficacy in CTCL cells through targeting p38γ. It is worth noting that in addition to FVP, other drugs targeting CDK9 also inhibit p38γ kinase activity, such as P276-00 and PIK75/F7 (Appendix A Figure 7); among them, FVP and F7 showed strong efficacy in CTCL (with IC_50_s of 120 nM and 29–33 nM, respectively). The mechanism of this synergy is proposed to be FVP targeted pathways of IL-21-BATF, p38 and NF-κB, which parallel that of niclosamide signaling.

Because we observed dual inhibition of p38γ and CDK9 by a single compound, FVP, it also suggests FVP harbors HDAC inhibitor property as well for it likens a synergistic effect of F7/PIK75, a potent TRAIL apoptosis sensitizer which through CDK9 inhibition with two HDAC inhibitors (SAHA and Abexinostat), respectively in CTCL. We demonstrated that the downstream targets of FVP have potential dual effects on the two kinases p38γ and CDK9, which resembles our previous studies of F7 in combination with the histone deacetylase inhibitor SAHA, evidenced by the loss of ITK, which parallels loss of p38δ and loss of STAT3 activation via phosphorylation by Western blot (Figure 3B). We observed a loss of ITK, a downstream biomarker of the TCR signaling pathway (Figure 3B). The interplay between ITK and STAT3 may direct the NFAT pathway, which is consistent with our Western blot result showing that DLGH1 reduction by FVP was blunted upon loss of p38γ (Figure 3D).

IL-21-BATF and its downstream proteins are also potential targets for therapeutic synergistic drug screening with p38 inhibitors. The IL-21 signaling pathway in T cells proceeds via binding of IRF4 to AP-1–IRF composite elements (AICEs), which requires involvement of the basic leucine zipper transcription factor ATF-like (BATF), thereby forming BATF–JUN–IRF4 complexes [29,33,34]. Thus, many target genes of IL-21 are regulated through BATF, JUN, IRF4, and STAT3 [33–37]. However, here we have observed at 24 hr FVP induced IL21 with decreased BATF (Figure 5C) which suggests alternative BATF-independent pathway by FVP upon IL21 induction via epigenetic regulations [38–40].

In addition, our Western blot results indicated that FVP targeted the STAT3–NF-κB pathway via p38 inhibition in CTCL Hut78 cells. The downregulation of both phosphorylation sites Ser32/36 and Ser178/180, the two critical sites of IκBα, together with pSTAT-Y705, further suggests that FVP-induced apoptosis occurs through IKKα regulation of NF-κB-dependent gene transcription via STAT3, which further led to apoptosis as the treatment time extended to 48 hr (Figure 3C). To trace FVP-induced apoptosis further upstream, it may occur through the IκBα protein, which we observed reduction of, alone and with its kinase IKKα/β. IKKα, an important IKK core element of the NF-κB cascade, regulates NF-κB-dependent gene transcription through a mechanism called “de-repression” of NF-κB target genes. IκB binds to NF-κB which keeps NF-κB in the inactive stage in the cytoplasm. In response to stimuli such as inflammatory cytokines, or various types of stress, IκB becomes phosphorylated on two critical serine residues. Phosphorylation of IκB at these two critical sites leads to its own poly-ubiquitination and its degradation; as a result, NF-κB is freed for nuclear translocation and activation of transcription. IKKα phosphorylates the SMRT repressor recruited by p50 and p52 homodimers, and promotes its nuclear export, together with HDAC3, and degradation [41]. Then IKKα phosphorylates NF-κB p65 on Ser536, which is chromatin-bound, to proceed to full transcription by acetylating p65 at Lys310 via p300 [42].

In summary, in this study we provided parallel pathways for synergistic drug development of FVP as a multi-target inhibitor in the light of p38 inhibitors in CTCL.

## Conclusions

We performed comprehensive assessments of important signaling proteins using western blot and kinomic profiling by the PamGene platform. We found that FVP affected CTCL cells at multiple layers of regulation, from gene regulation to post-transcriptional and post-translational regulation. In Western blots, we observed that ITK and p-DLGH1 S158 were downregulated by FVP treatment, which indicates that the NFAT pathway was blocked via p38γ inhibition. In the PTK panel of the PamGene platform, we observed an important role for p-Cam Y100 and p-CD3ζ at Y123 and Y153, which were influenced by FVP upon 4 hr treatment. In the STK panel, we observed that the majority of protein phosphorylation at Ser/Thr was decreased, which included p53, Rel, and PTK6. We also performed analyses using the TRANSFAC and JASPAR databases, in which eight proteins showed reduced phosphorylation; these proteins are subject to NF-κB transcriptional regulation, because they all harbor NF-κB binding motif(s) in their promoter regions. We are also actively investigating epigenetic regulation through Histone deacetylase (HDAC) or methyltransferases (DNMT1) by FVP. To select drugs that have synergistic effects with FVP, we must consider the side effects of a higher dose FVP, which impairs human ion channel complexes. Using knowledge gained from the present study on signaling pathways that are complementary to FVP p38γ inhibition, we have since designed experiments to select candidate drugs that show synergy with FVP; we identified the FDA-approved drug niclosamide as an ideal match for FVP, which we will investigate in future *in vivo* studies.

## Materials and Methods

### Compounds, samples, and cell culture

FVP and niclosamide were ordered from Selleck. Cpd 4f was ordered from Sigma. DMSO was used as solvent for FVP, cpd 4f, and niclosamide. Compounds in solution were aliquoted and stored at −80°C until use. Isolation of peripheral blood mononuclear cells from healthy donors, and culture of CTCL cell lines (Hut78) were performed as previously described [43].

### Viability assays using trypan blue exclusion and CellTiterGlo Cell Viability Assay

Cell viability was calculated by diluting cell suspensions 1:1 in 0.4% Trypan Blue solution (Sigma, St. Louis, MO) and counting the number of viable cells using a TC20^™^ automated cell counter (Bio-Rad, Irvine, CA) which automatically excludes the number of non-viable cells stained with trypan blue per total cells. CellTiterGlo Cell Viability Assay (Promega) method was used as described previously [5]. All data points are an average of triplicate experiments.

### *In vitro* kinase assay using ADP-Glo

To identify p38γ inhibitors using *in vitro* kinase assay, a library of kinase inhibitors was screened. The library consists of 244 compounds on three plates (EMD Cat#539744, #539745 and #539746) that are mostly ATP mimics. All compounds are cell-permeable, reversible, and well-characterized. For biochemical screening, *in vitro* kinase assays were performed using an ADP-Glo kit (Promega, Madison, WI) according to the manufacture’s protocol. All data points are average of triplicate experiments unless stated otherwise, and all compounds have been tested to show no inhibition of luciferase activities when using the ADP-Glo kit. Briefly, for *in vitro* assay experiments, human recombinant p38α, β, γ, or δ protein kinases (active full-length) were acquired from SignalChem, and the assay was performed followed protocols from the company. The p38 kinase was pre-incubated with FVP in a dose-dependent manner for 10 min before synthetic peptide substrates (IPTTPITTTYFFFKKK) were added at final concentration of 0.2 μg/μL followed by addition of ATP. Then, ADP-Glo Reagent was incubated in the mixture at room temperature for 40 min, followed by incubation of Kinase Detection Reagent for another 30 min. IC_50_ values were determined using CalcuSyn software (Biosoft, Cambridge, United Kingdom).

### Enzyme kinetics

The inhibitory mechanism of FVP was measured using the TR-FRET method [44]. Assays were conducted in a 384-well black round-bottom plate in kinase reaction buffer (50 mM HEPES, pH 7.5; 10 mM MgCl2; 1 mM EGTA, 100 μM Na3VO4; 0.01% Tween-20; 0.5 mM DTT). p38γ kinase (700 ng/ml) was mixed with ULightTM-4E-BP1 peptide (50 nM, PerkinElmer, Waltham, MA) and varying concentrations of ATP (1, 1.5, 2, 3, 4, 6, 15, 30 μM) and FVP (0, 50, 200, 400, 1000 and 2000 nM). Time course data were collected by stopping the kinase reaction at various times by adding detection buffer containing Europium-anti-phospho-4E-BP1 antibody (4 nM, PerkinElmer). Fluorescence signals were measured at 665 nm with a 50 μs delay after excitation at 320 nm using a CLARIOstar microplate reader. The signal ratio at 665/620 nM was used for data analysis. The inhibition mechanism and kinetic rate constants were analyzed using GraphPad Prism 7 software (GraphPad, La Jolla, CA).

### Apoptosis detection using Annexin V antibody FITC staining

Hut78 cells, seeded at 2 × 10^5^ cells/ml, were treated with FVP (0, 60 nM, 120 nM, 240 nM, or 480 nM) for 48 hr. The BD FITC Annexin V Apoptosis Detection Kit was used according to the manufacturer’s protocol. Briefly, cells were collected and washed with 1x PBS twice, resuspended in 1x binding buffer, and incubated with FITC Annexin V antibody and propidium iodide for 15 minutes in the dark at room temperature. Samples were analyzed using an Attune Nxt cytometer within 1 hour.

### NMR studies

2D; Ileδ1-[13CH3]; Leu, Val - [13CH3, 12CD3]; Metε--[13CH3]-labeled p38γ sample was prepared, and the NMR spectra were collected and analyzed as described [5]. In the complex sample, 100 μM was added with five-fold of FVP. NMR chemical shift changes were calculated as

$$\text{CSP}=\surd \left(\Delta \delta 〗{\;}_{-}{\text{H}}^{\wedge }2+〖{\left(\text{0}.154\cdot \Delta \delta_{-}\text{N}\right)}^{\wedge }2+{\left(\text{0}.341\cdot 〖\Delta \delta 〗{\;}_{-}\text{C}\right)}^{\wedge }2\right)$$

where ΔδH, ΔδN, and ΔδC are the chemical shift differences between the free and bound states in the proton, nitrogen, and carbon dimensions, respectively.

#### CSP normalization method:

The CSP for each compound was normalized separately. The values in each dataset (different compounds) were between 0 and 1. CSPs were calculated and further normalized using the min-max method using the following equation: normalized CSP = (CSP - CSPmin)/(CSPmax - CSPmin); where CSPmax and CSPmin are the largest and smallest CSPs in each titration of the compound.

### IC_50_ value determination of cytotoxicity of FVP against Hut78 CTCL cells

To determine the cytotoxicity of FVP in cancer cell line panel assay, MTS assays (Promega) were performed and cell viability was determined as described previously [45]. CellTiterGlo Cell Viability Assay (Promega) was used to measure viability of Hut78 CTCL cells. Absorbance was monitored at 490 nm using an automated BMG PHERAstar plate reader (BMG Labtech, Ortenberg, Germany). IC_50_ values were determined using CalcuSyn software. All experiments were repeated in three independent experiments and data represented are the average of triplicate experiments.

### Western blot

Western blots were performed as described previously [43]. Rabbit primary antibodies (Cell Signaling Technology (CST), Danvers, MA) were used at the following dilutions: anti-p38α, -p38β, -p38γ, and -p38δ (1:1000), anti-ß-tubulin (1:2000), anti-p38 GAPDH (1:1000); anti-β-Actin (8H10D10), mouse mAb (1:2000). Anti-DLGH1 and anti-phosphor DLGH1 at serine 158 and 431, total DLGH1 (SAP97) are affinity-purified sheep polyclonal antibodies which were from University of Dundee, Scotland (1:1000). HRP-conjugated goat anti-rabbit (Cat#7074) and anti-mouse IgG, HRP-linked Antibody Cat#7076 (CST, 1:2000) were used as secondary antibodies.

Primary antibodies purchased from CST were used at the following dilutions: p38α (#9218, 1:1000), p38β (#2339, 1:1000), p38δ (#2308, 1:1000), p38γ (#2307, 1:1000), p-p38 Thr180Tyr182 (#4511, 1:1000), GAPDH (#2118, 1:5000), CBP (#7389, 1:1000), p300 (#86377, 1:1000), Ezh2 (#5246, 1:1000), TNFα (#3707, 1:1000), and Anti-SAPK3 (p38γ, ab205926) were purchased from abcam and used at 1:1000 dilution. p-p38 Tyr323 antibody (#12322-1) was purchased from Signalway Antibody and used at 1 μg/mL.

### Kinase array profiling using PamGene technology

The kinase activity profiling assay was performed using the PamChip®4 microarray utilized by PamStation®12. The PamChip®4 kinase array platform monitors phosphorylation of 144 Tyr or 144 Ser/Thr kinase peptide substrates manufactured with 3-D membranes. The experiment was carried out using the vendor’s protocol (PamGene, Netherlands). Briefly, Hut78 CTCL cells were treated with 0.12 μM and 2 μM FVP for 4 hr or 24 hr. DMSO was used as a vehicle control. Intact whole cell lysates were prepared using M-PER Mammalian Extraction Buffer supplemented with Halt Phosphatase and Protease Inhibitor Cocktail (Pierce, Waltham, MA). PamChip®4 arrays were blocked with 2% bovine serum albumin (BSA). Cell lysates (5 μg/array for Tyr chip and 3 μg/array for Ser/Thr chip) were applied per array of PamChip®4. Next, a kinase reaction mixture (KRM) containing kinase buffer, DTT, kinase additives, BSA, fluorescent antibody, and 400 μM final concentration of ATP was added to each array. Phosphorylation of kinase activities was detected with a fluorescent-conjugated antibody and images were acquired by an optical CCD-camera equipped in the PamStation®12. Each experiment was performed in triplicate. The data was analyzed with the advanced software BioNavigator62.

### p38γ kinase assay *ex vivo* using PamGene kinomic array platform

To determine whether FVP inhibits phosphorylation of p38γ substrates, we performed the kinase activity profiling assay *ex vivo* using PamChip microarrays (PamGene, Netherlands). The kinase array platform monitors phosphorylation of 144 Ser/Thr kinase peptide substrates in the STK chip manufactured with 3-D membranes. The experiment was carried out using the vendor’s protocol with a minor modification. Briefly, STK PamChips were blocked with 2% BSA. For the basic mixture solution, BSA, STK antibody mix, and ATP were mixed in the kinase buffer. Next, for the reaction, recombinant full-length p38γ kinase protein (50 ng/well) obtained from SignalChem (BC, Canada) and ATP were added to the basic mixture solution, followed by addition of DMSO or FVP (0.5 μM, 1 μM, and 2 μM at final concentrations). The final concentration of ATP was 100 μM and reaction volume was 40 μL in each well of chips. After 90 min reaction, a fluorescent-conjugated (FITC) antibody was added to the reaction mixture. Each reaction was performed in triplicate. Images of phosphorylation of kinase activities were acquired by an optical CCD-camera equipped in the PamStation 12. The data was analyzed with the advanced software BioNavigator62.

### Statistical analysis

All experimental data are shown as mean ± SEM unless indicated otherwise. The statistical significance of differences, i.e., in cell viability assays, were assessed by student T-test (SPSS, IBM, Armonk, NY) or one-way ANOVA (GraphPad PRISM v. 3.0, GraphPad). Differences were considered significant if P < 0.05. Sign Test, a nonparametric test, was used for inhibitory IC_50_ of 8 cells of NCI 60 cells by p38γ candidates, significant if P<0.005.

## Supplementary Material

JCH-21-028-Appendix

## Figures and Tables

**Table 1:** Completed clinical trials of Flavopiridol (Alvocidib) with results posted.

Clinical Trial	Phase	Intervention	Results
NCT00445341	I/II	Study of **flavopiridol** in relapsed or refractory mantle cell lymphoma (MCL) and diffuse large B-cell lymphoma (DLBCL).	2/26 partial response, no complete response, 14/28 experienced serious adverse events
NCT00112723^1^	I/II	Study of side effects and best dose of **flavopiridol** and to see how well it works in treating patients with lymphoma or multiple myeloma.	6/43 partial response
NCT00058240	I/II	Study of side effects and best dose of **flavopiridol** in treating patients with previously treated chronic lymphocytic leukemia or lymphocytic lymphoma.	ORR 21/52, serious adverse events: 11/42 in lower dose group, and 1/16 in highest dose group
NCT00331682	II	Study of how well giving docetaxel followed by **flavopiridol** works in treating patients with refractory metastatic pancreatic cancer.	3/9 stable disease, with median 8 weeks to disease progression, and median overall survival 4.2 months. 9/9 experienced serious adverse events
NCT00083122	II	Study of how well giving cisplatin together with **flavopiridol** works in treating patients with advanced ovarian epithelial cancer or primary peritoneal cancer	1 complete response, 8 partial responses out of 45 patients. Median OS 17.5 months, median time to progression 4.3 months. 23/45 experienced serious adverse events
NCT00991952	II	Study of how well giving irinotecan hydrochloride with or without **alvocidib** works in treating patients with advanced stomach or gastro-esophageal junction cancer that cannot be removed by surgery	1/13 partial response, 4/13 stable disease. 6/13 with serious adverse events
NCT00407966	II	Study of side effects and how well giving **alvocidib** together with cytarabine and mitoxantrone works in treating patients with newly diagnosed acute myeloid leukemia.	45/45 complete response. 19/45 with serious adverse events
NCT01349972	II	Study of how **alvocidib**, cytarabine, and mitoxantrone hydrochloride work compared to cytarabine and daunorubicin hydrochloride in treating patients with newly diagnosed acute myeloid leukemia.	76/109 complete response. 53/114 serious adverse events
NCT00957905	II	Study of **alvocidib** and oxaliplatin to see how well they work when given with or without fluorouracil and leucovorin calcium in treating patients with relapsed or refractory germ cell tumors.	2/7 stable disease for Flavo+Oxaliplatin (4/7 serious adverse effect). For Flavo +FOLFOX, 6/25 partial response, 10/25 stable disease. (7/29 serious adverse effect)
NCT00795002	II	Study of two different schedules of **alvocidib** to compare how well they work when given together with cytarabine and mitoxantrone in treating patients with newly diagnosed acute myeloid leukemia.	Complete response: 24/37 (arm 1), 29/37 (arm 2). Serious adverse event: 8/39 (arm 1), 8/39 (arm 2)
NCT00634244	II	Study to compare three different combination chemotherapy regimens to see how well they work in treating patients with relapsed or refractory acute myeloid leukemia.	10/36 complete remission. 36/36 serious adverse event

1 Terminated (Administratively Complete).

## Abbreviations:

FVP: Flavopiridol
CDK: Cyclin-dependent Kinases
CTCL: Cutaneous T-cell lymphoma
FDA: Food and Drug Administration
NMR: Nuclear Magnetic Resonance
CSP: Chemical Shift Perturbation
STAT: Signal Transducer and Activator of Transcription
NFAT: Nuclear Factor of Activated T-cells
ITK: Interleukin-2-inducible T-cell Kinase
IKK: Inhibitor of nuclear factor Kappa-B Kinase
NF-κB: Nuclear Factor kappa-light-chain-enhancer of activated B cells
DLGH: Discs Large Homolog
PTK: Protein Tyrosine Kinase
STK: Serine-threonine kinase
PI3K: Phosphoinositide 3-kinase
TCR: T-cell Receptor
PTK6: Protein Tyrosine Kinase 6
HDAC: Histone Deacetylase
