# Supplementary material for: Flavopiridol (Alvocidib), a Cyclin-dependent Kinases (CDKs) Inhibitor, Found Synergy Effects with Niclosamide in Cutaneous T-cell Lymphoma
Source: J Clin Haematol. Author manuscript; Available in PMC 2021 Jul 1. (PMC8248901; doi:10.33696/haematology.2.028)
Supplement: JCH-21-028-Appendix [file NIHMS1716549-supplement-JCH-21-028-Appendix.zip › JCH-21-028-Appendix.docx]

Appendix A: Supplemental Figures


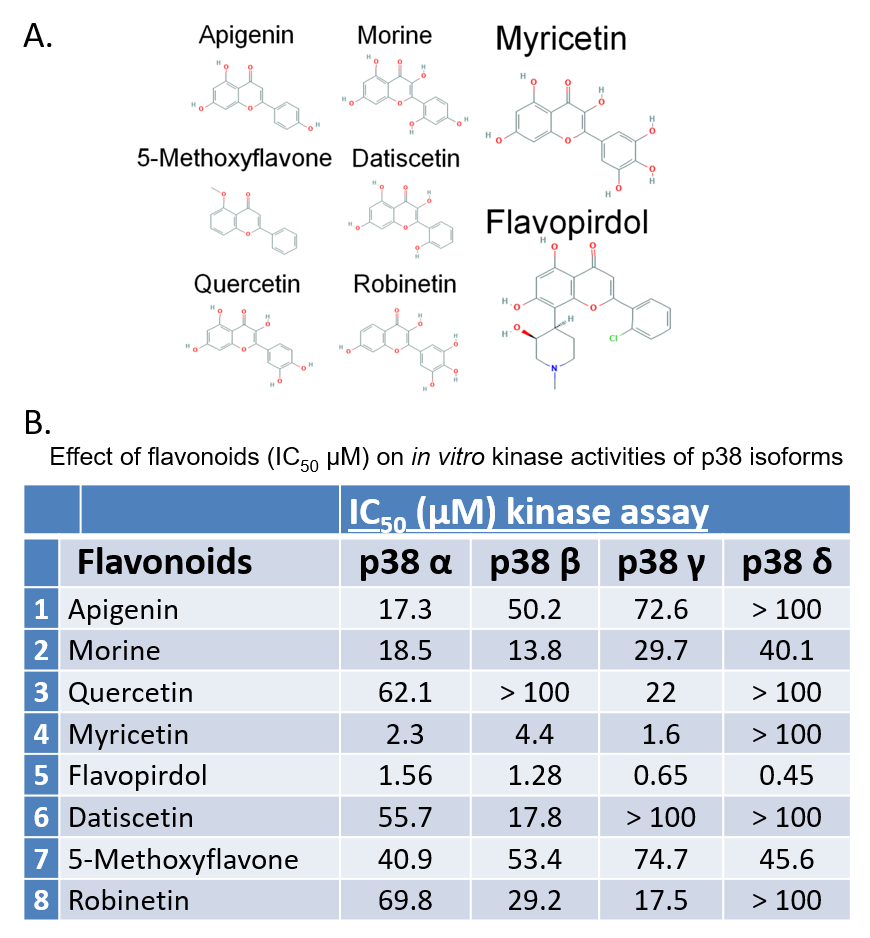


**Figure 1.**

**Appendix A. Figure 1.** (A). Structure of indicated flavonoid compounds; (B). Effect of indicated flavonoids (IC_50_ µM) on in vitro kinase activities of p38 isoforms.


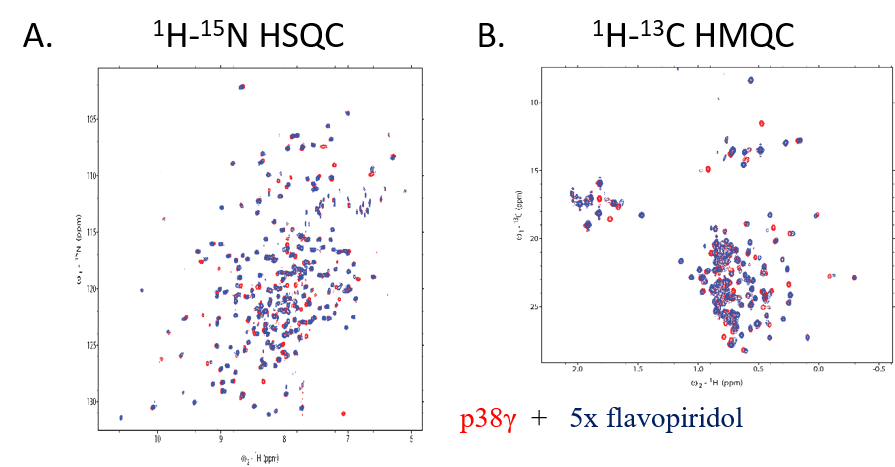


**Appendix A. Figure 2.** Extensive NMR chemical shift changes and line broadening were observed in both (A) 1H-15N HSQC and (B) 1H-13C HMQC spectra upon the addition of FVP to p38γ.


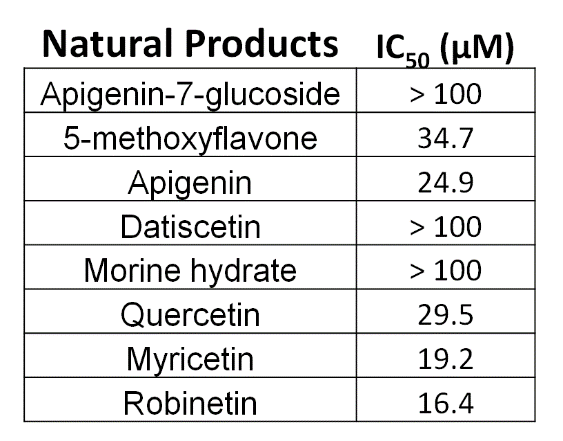


**Appendix A. Figure 3.** Comparison of cytotoxicity of FVP (IC_50_= 0.094 µM) to other flavonoid compounds in Hut 78 cells. FVP was the far more potent to Hut78 cells than other flavor-backbone compounds such as myricetin with IC_50_= 19.2 µM.


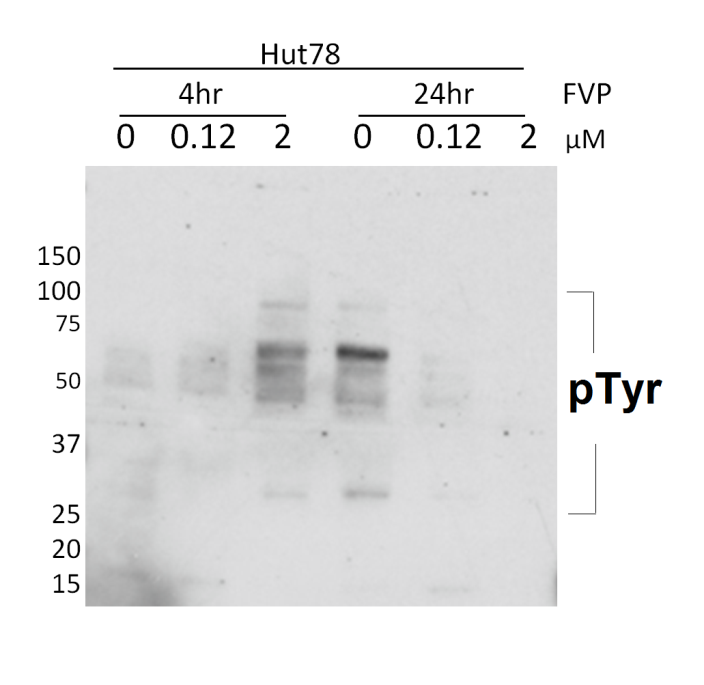


**Appendix A. Figure 4.** Proteins phosphorylated by Tyrosine kinases are increased at 4hr but decreased at 24hr of FVP treatments by western blot of p-Tyr antibody staining of cell lysates of Hut78 cell with FVP of two dosages at 2 time points, 4hr and 24hr.


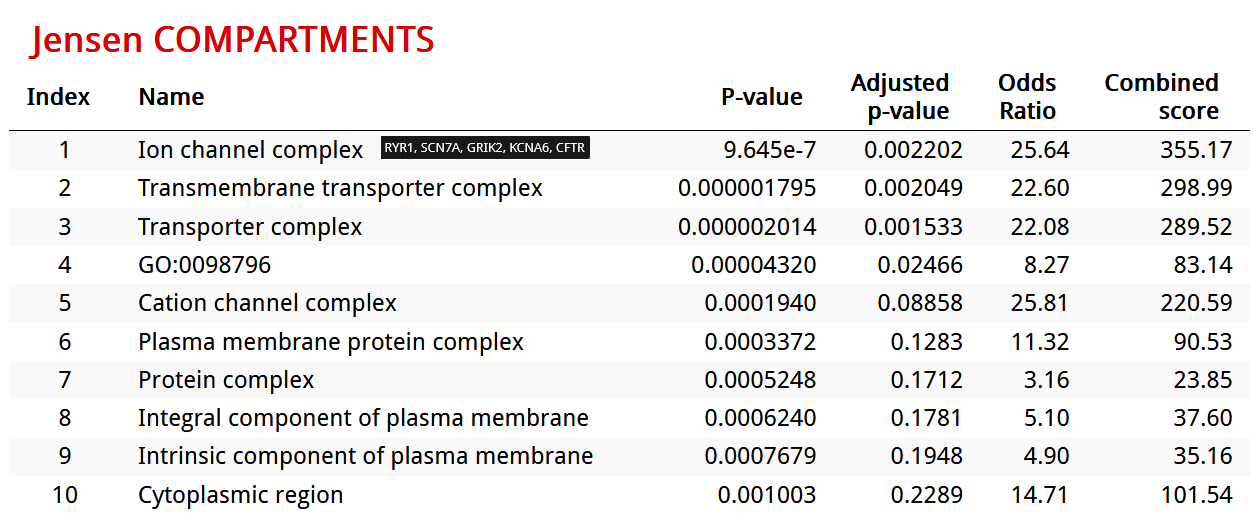


**Appendix A. Figure 5.** A list of 15 proteins that showed differential expressed with two dosages of FVP treatment 2uM and 120nM in pamgene analysis followed by Jensen compartments (EnrichR) analysis. The Top hits in Jensen compartments is Ion channel complex with a p-value =9.645e-7, of 5 proteins similarity of RYR1, GRIK2, SCN7A, KCNA6 and CFTR on the list which implicates a notion that FVP cause damage on the Ion channel complex, most likely Calcium channel when the concentration is higher than 240nM.


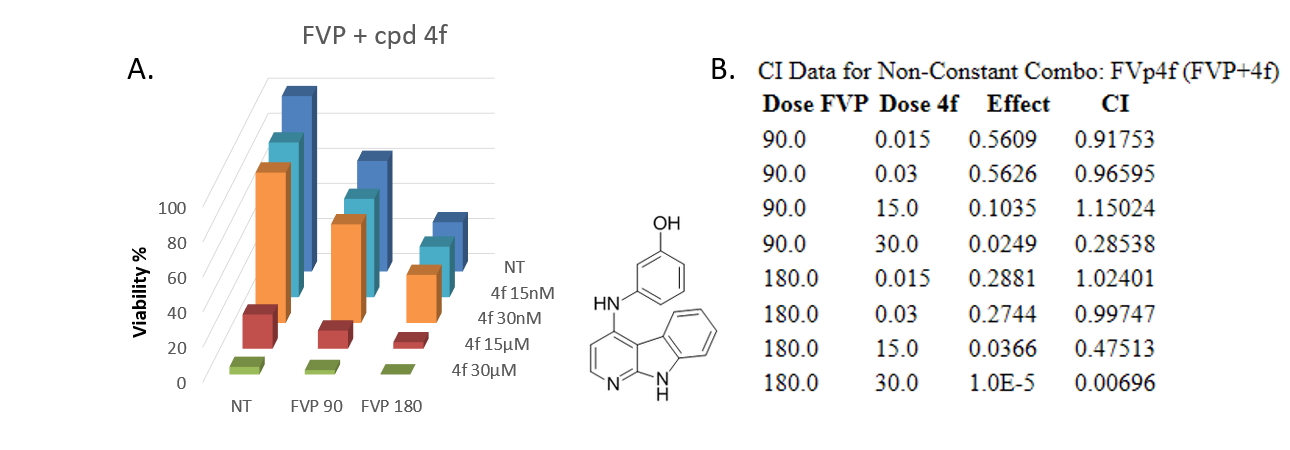


**Appendix A. Figure 6.** Determination if a PTK6 inhibitor, cpd4f is FVP synergistic drug by cell viability assays. (**A**) Cell viability assay of Hut78 cells with increasing dosages of FVP; and cpd4f. (**B**) CI data calculation for FVP and cpd4f.


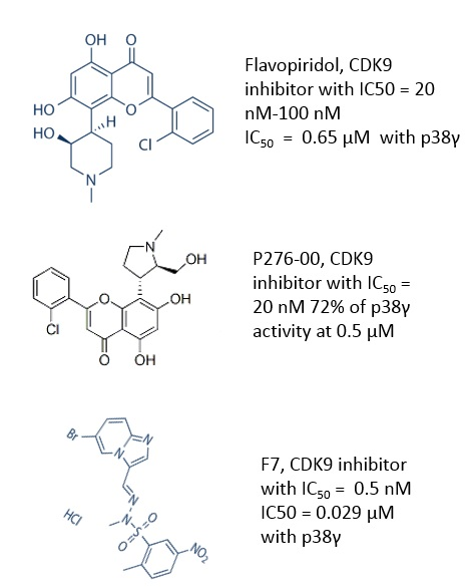


Appendix A. Figure 7. FVP is among the drugs that are dual inhibitors of both p38g and CDK9, p276-00 and F7/PIK75.
